# Pulmonary embolism in a patient with eltrombopag-treated aplastic anaemia and paroxysmal nocturnal haemoglobinuria clone during COVID-19 pneumonia

**DOI:** 10.1186/s12959-022-00407-w

**Published:** 2022-08-24

**Authors:** Bosi Alessandro, Barcellini Wilma, Fattizzo Bruno

**Affiliations:** 1 Haematology Unit, Fondazione IRCCS Ca’ Granda Ospedale Maggiore Policlinico, Via Francesco Sforza 35, 20100 Milan, Italy; 2 Department of Oncology and Haemato-oncology, University of Milan, Milan, Italy

**Keywords:** Aplastic anemia, Paroxysmal nocturnal hemoglobinuria, COVID-19, Eltrombopag, Thrombosis

## Abstract

Thrombosis in patients with thrombocytopenia has several risk factors, both disease-related and treatment-associated. Recently, COVID-19 infection was recognized as an additional risk factor, further complicating the delicate balance between thrombosis and bleeding in these patients. Here we describe the case of a patient with aplastic anaemia on eltrombopag who developed pulmonary embolism during COVID-19 pneumonia, despite receiving oral anticoagulation with edoxaban. Notably, he was also carrying a large paroxysmal nocturnal haemoglobinuria clone, although without evidence of haemolysis. The presented case recapitulates some of the open questions in thrombotic risk management of cytopenic patients, such as the management of thrombopoietin receptor agonists and the choice of anticoagulation in PNH, while also accounting for the additional thrombotic risk linked to COVID-19.

## Background

COVID-19 has been associated with an increased incidence of thrombosis and pulmonary embolism [1, 2], even in patients under anticoagulation [3]. This adds to the several known risk factors for thrombosis, complicating the management of haematological conditions with an instable haemostatic balance. Here we report the case of a patient with severe aplastic anaemia (SAA) carrying a paroxysmal nocturnal haemoglobinuria (PNH) clone which is a known thrombophilic condition [4]. The patient developed a major thrombotic event during COVID-19 infection, despite direct oral anticoagulation (DOAC). Additionally, he was receiving a thrombopoietin receptor agonist (eltrombopag), which has been associated with an increased thrombotic risk in patients with immune thrombocytopenia (ITP) [5].

## Case presentation

**Fig. 1:**
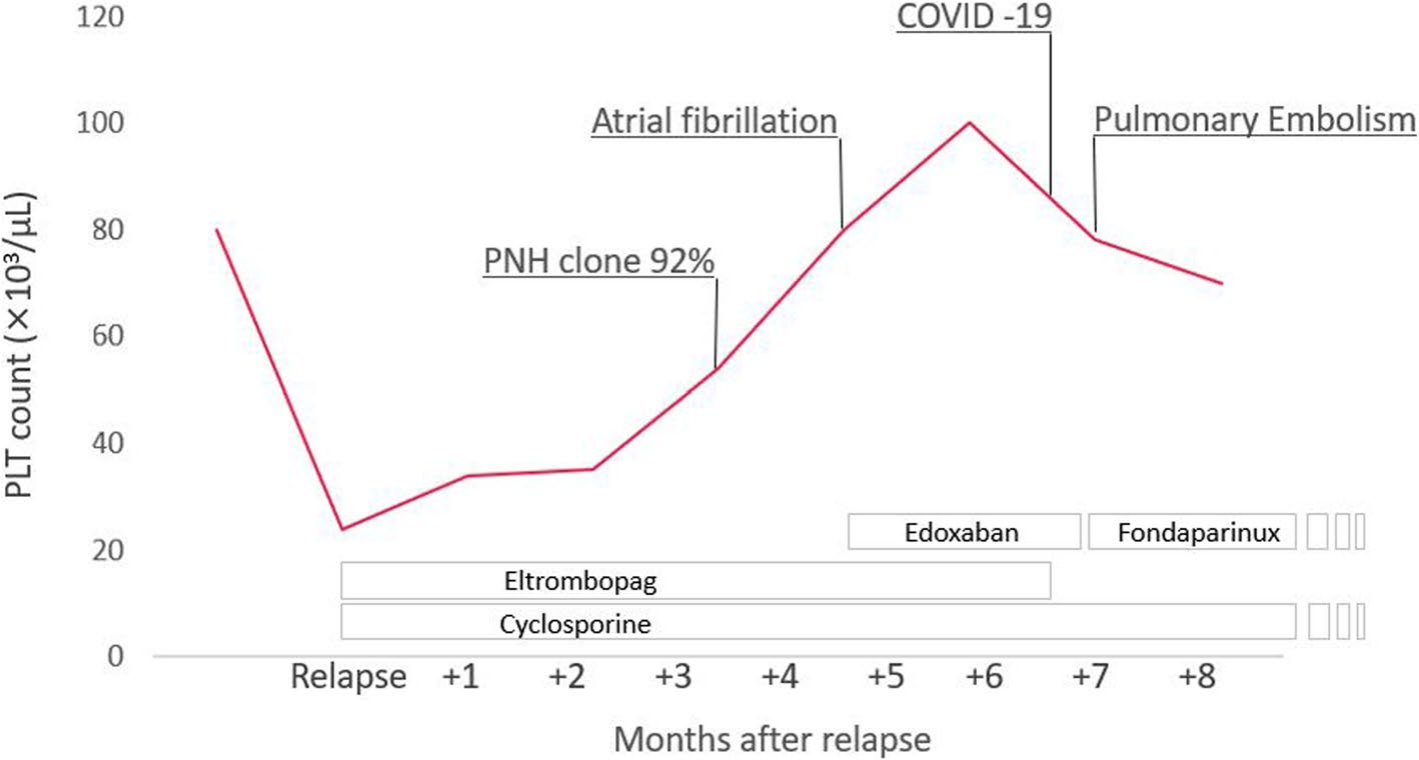
Clinical course of the presented case over time. Platelet counts, treatment and clinical events are shown since relapse of Aplastic Anaemia in June 2021 to COVID-19 infection

**Fig. 2:**
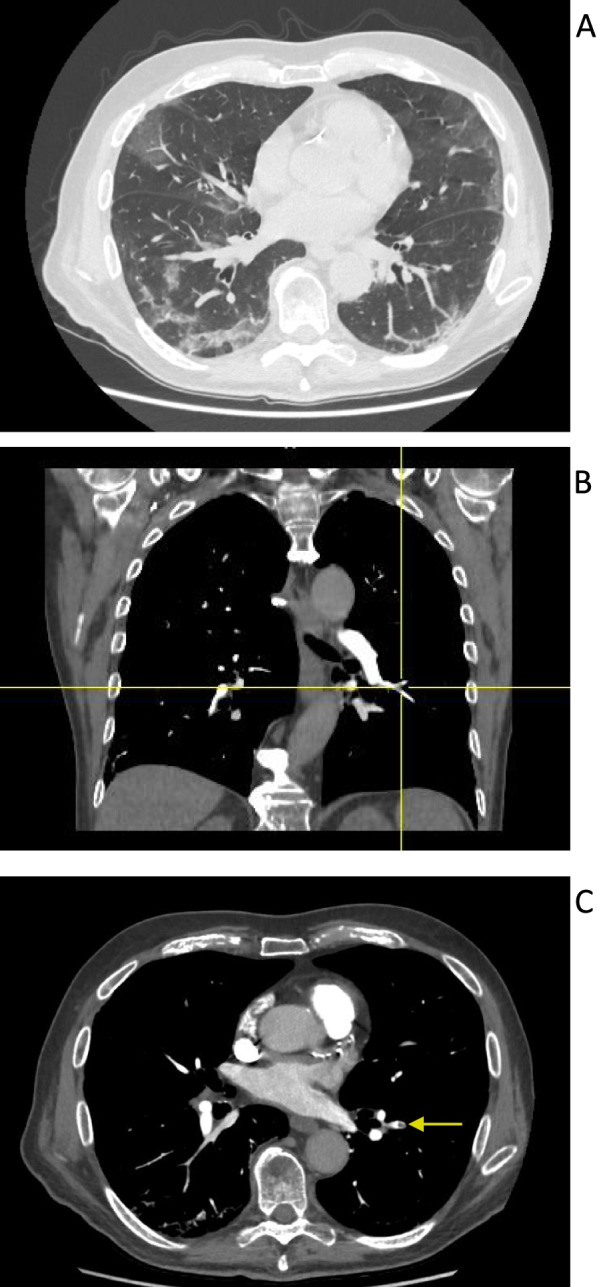
thorax contrast-enhanced CT scan images at admission. **A** Bilateral ground glass opacities, indicative of interstitial pneumonia. **B** and **C** Arterial phase scans indicating a filling defect of the left anteromedial segmental basal artery (pointer in **B**. and arrow in **C**)

A 74-year-old Caucasian man was diagnosed with SAA and treated with antithymocyte globulin, cyclosporine A and corticosteroids, which was followed by complete remission. Oral prednisone and cyclosporine were slowly tapered off until complete interruption after 3 years from diagnosis. Seven years later, due to worsening thrombocytopaenia (platelets, PLT: 24 × 10^3^/μL), a bone marrow trephine biopsy was repeated, showing hypocellularity (15–20%), absence of megakaryocytes and a polyclonal T-cell infiltrate (15% of cellularity). A relapse of aplastic anaemia was diagnosed and oral cyclosporine (2.5 mg/kg day), combined with low-dose prednisone and eltrombopag (150 mg/day after initial up-titration) were initiated, leading to a progressive increase of PLT count (Fig. 1). Additionally, fluorescent aerolysin (FLAER) analysis on peripheral blood revealed a large PNH clone (92% on granulocytes and monocytes), which was absent at diagnosis. Parameters of haemolysis were only mildly altered, with a serum lactate dehydrogenase (LDH) level of 1.19 x the upper limit of normality (ULN) and total bilirubin level of 1.19 mg/dL, along with mild anaemia (Hb 12.5 g/dL), so that complement inhibition was not started. Likewise, prophylactic anticoagulation was not started, also considering the low PLT value (about 50 × 10^3^/μL). Four months after relapse, the patient developed atrial fibrillation and began oral anticoagulation with edoxaban 60 mg/day (PLT values were permissive). Two months later, he developed COVID-19 (positive PCR-based nasopharyngeal swab) requiring access to the Emergency Room due to fever and respiratory failure. Notably, he was not vaccinated against SARS-CoV-2. At time of presentation, Hb was 13.5 g/dL, PLT were 77 × 10^3^ /μL, WBC were 6.12 × 10^3^ /μL, with 5.37 × 10^3^ /μL neutrophils, whereas LDH was 341 U/L (1.39 times ULN). A contrast-enhanced CT-scan revealed bilateral ground glass opacities and a filling defect of the left anteromedial segmental basal artery, indicative of pneumonia and pulmonary embolism (Fig. 2). Respiratory failure appeared to be more related to pneumonia than to pulmonary embolism, considering the extension of lung opacities and, conversely, the non-massive nature of the embolism. Low flow oxygen therapy was started and subsequently escalated to continuous positive airway pressure (CPAP) ventilation. A transthoracic echocardiography showed a normal ejection fraction with a Pulmonary Artery systolic Pressure (PAPs) of 25 mmHg. Edoxaban was interrupted and replaced by subcutaneous fondaparinux (7.5 mg/day), whereas eltrombopag was discontinued. No antiviral treatment for COVID-19 was administered. His general conditions progressively improved, until interruption of non-invasive ventilation and oxygen therapy. Notably, PLT counts remained stably > 50 × 10^3^/μL throughout hospital stay and no transfusion was needed. At last follow-up, 2 months past COVID-19 infection, the patient had stable blood counts (Hb 12 g/dL, WBC 5.26 × 10^3^/μL, PLT 92 × 10^3^/μL, absolute reticulocyte count 76 × 10^3^/μL) and was counselled to continue fondaparinux. Of interest, thrombophilia screening, including antiphospholipid antibodies, was negative.

## Discussion and conclusions

The presented case is paradigmatic of the complex interplay of risk factors that can lead to thrombosis in a haematological patient experiencing COVID-19. It also raises questions on how to properly manage eltrombopag and anti-coagulation in such an uncharted territory. Regarding eltrombopag, manufacturer’s brochure advises a dose reduction or interruption in case of thrombocytosis (i.e. PLT > 250 × 10^3^ /μL) (https://www.ema.europa.eu/en/documents/product-information/revolade-epar-product-information_en.pdf), whilst no advice is given in case of thrombosis. The risk-benefit ratio of discontinuing eltrombopag should be weighed on a case-by-case basis, considering PLT count stability, risk of rebound thrombocytopenia and consequent probability of bleeding. In addition, no clear-cut indications are provided by the recently published guidelines for ITP [6].

Similarly, there is lack of guidance on how to manage eltrombopag during COVID-19 infection, which is a recognized thrombophilic condition. In ITP, published guidelines do not advise eltrombopag discontinuation and suggest prophylactic heparin in hospitalized patients with permissive PLT values [7, 8]. In our case, eltrombopag was interrupted after diagnosis of pulmonary embolism; nonetheless, the patient maintained stable and safe PLT counts.

Another open question is the role of complement inhibition in preventing thrombosis in patients with large PNH clones, yet without ongoing haemolysis [9]. In our case, the presence of a large PNH clone, together with the occurrence of a thrombotic event, may have warranted the initiation of life-long complement inhibition. However, the patient had neither evidence of anaemia nor of significant haemolysis, except a mild LDH elevation, possibly confounded by severe COVID-19 pneumonia. Therefore, the specific contribution of haemolytic PNH to thrombosis was questionable. Additionally, considering timing and site (pulmonary arteries), the thrombotic event was considered as triggered by COVID-19, hence by a transient cause, and the decision to start complement inhibition was deferred. Beyond the specific case, it should be noted that a clear reduction of thrombotic risk in PNH has been demonstrated only by anti-complement treatment [10], while the role of primary thromboprophylaxis is still controversial [11, 12].

A further consideration is that, in our patient, thromboembolism was not prevented by the therapeutic doses of edoxaban, which was in fact replaced by fondaparinux. In this respect, a question arises as whether PNH patients who are therapeutically anticoagulated or are receiving thromboprophylaxis with a DOAC should be switched to therapeutic heparinoids during COVID-19 infection, before they develop overt thrombosis. This approach should be considered, particularly in patients requiring hospital admission, and may be supported by the putative anti-inflammatory role of heparinoids [13], which may be relevant for COVID-19 related thromboinflammation. As a matter of fact, patients with COVID-19 who required hospital admission had better outcomes if started on therapeutic heparins [14], which were also shown to interfere with SARS-CoV-2 infection [15], while no similar evidence is available for DOACs. Additionally, heparinoids may also exert a modulatory effect on complement cascade, supporting their use in PNH-related thrombosis. Poor evidence exists in this setting about the efficacy of DOACs [16–18], that also proved inferior in patients with anti-phospholipid syndrome [19, 20].

In conclusion, our case highlights that the management of thrombosis in patients with several predisposing conditions (large PNH clone, eltrombopag, COVID-19) is challenging. It also advocates for future studies to clarify the role of DOACs in subjects with multiple risk factors, including PNH.

## Data Availability

Not applicable.

## Abbreviations

CPAP: Continuous positive airway pressure
DOAC: Direct oral anticoagulation
ITP: Immune thrombocytopaenia
Hb: Haemoglobin
PLT: Platelet
PNH: Paroxysmal nocturnal haemoglobinuria
SAA: Severe aplastic anaemia
WBC: White blood cells
